# Gyrification Connectomes in Unmedicated Patients With Schizophrenia and Following a Short Course of Antipsychotic Drug Treatment

**DOI:** 10.3389/fpsyt.2018.00699

**Published:** 2018-12-20

**Authors:** Eric A. Nelson, David M. White, Nina V. Kraguljac, Adrienne C. Lahti

**Affiliations:** ^1^Department of Psychology, University of Alabama at Birmingham, Birmingham, AL, United States; ^2^Department of Psychiatry, University of Alabama at Birmingham, Birmingham, AL, United States

**Keywords:** schizophrenia, antipsychotic medication, graph analysis, gyrification index, prospective design

## Abstract

Schizophrenia (SZ) is a d isease characterized by brain dysconnectivity and abnormal brain development. The study of cortical gyrification in schizophrenia may capture underlying alterations reflective of neurodevelopmental abnormalities more accurately than other imaging modalities. Graph-based connectomic approaches have been previously used in schizophrenia to study structural and functional brain covariance using a diversity of techniques. The goal of the present study was to evaluate morphological covariance using a measure of local gyrification index in patients with schizophrenia. The aims of this study were two-fold: (1) Evaluate the structural covariance of local gyrification index using graph theory measures of integration and segregation in unmedicated patients with schizophrenia compared to healthy controls and (2) investigate changes in these measures following a short antipsychotic drug (APD) treatment. Using a longitudinal prospective design, structural scans were obtained prior to treatment in 34 unmedicated patients with SZ and after 6 weeks of treatment with risperidone. To control for the effect of time, 23 matched healthy controls (HC) were also scanned twice, 6 weeks apart. The cortical surface of each structural image was reconstructed and local gyrification index values were computed using FreeSurfer. Local gyrification index values where then parcellated into atlas based regions and entered into a 68 × 68 correlation matrix to construct local gyrification index connectomes for each group at each time point. Longitudinal comparisons showed significant group by time interactions for measures of segregation (clustering, local efficiency) and modularity, but not for measures of integration (path length, global efficiency). *Post-hoc* tests showed increased clustering, local efficiency, and modularity connectomes in unmedicated patients with SZ at baseline compared to HC. *Post-hoc* tests did not show significant within group differences for HCs or patients with SZ. After 6 weeks of treatment, there were no significant differences between the groups on these measures. Abnormal cortical topography is detected in schizophrenia and is modified by short term APD treatment reflective of decreases in hyper-specialization in network connectivity. We speculate that changes in the structural organization of the brain is achieved through the neuroplastic effects that APDs have on brain tissue, thus promoting more efficient brain connections and, possibly, a therapeutic effect.

## Introduction

Schizophrenia is a disease characterized by brain structural (1) and functional dysconnectivity (2), abnormalities of gray and white matter (3) as well as evidence of abnormalities in brain development (4–6). Primary cortical folding of the human brain begins as early as the 8th week of gestation with secondary folding having formed by the third trimester and tertiary folding patterns continuing into the postnatal period (7). As a marker of early neurodevelopmental abnormalities, the study of cortical gyrification through the use of MRI technology has been effective in differentiating between preterm infants with and without brain injury as well as between term infants and both preterm infants with and without brain injury scanned at equivalent postnatal age (8). For these reasons, the study of cortical gyrification in schizophrenia might be able to capture underlying alterations reflective of neurodevelopmental abnormalities better than other imaging modalities. Previous studies have reported altered gyrification patterns among subjects with high genetic risk of schizophrenia (9), first-degree relatives (10), first-episode psychosis patients (11), as well as patients with chronic schizophrenia (12, 13). However, increases and decreases in gyrification have been observed across illness phases and studies. These inconsistent findings are in part the result of different methods being used for measuring gyrification, investigation of different brain regions, and the study of patients at various stages of illness. Further indicative of its ability to index neurodevelopment abnormalities, cortical gyrification in schizophrenia has been linked to the presence of neurological soft signs (14), shown a better discriminatory ability to identify patients with more severe illness than cortical thickness (15), and identified greater gyrification abnormalities in those not responding well to treatment (16).

Graph-based connectomic approaches have been used in schizophrenia to study brain structural and functional covariance using a diversity of techniques including electroencephalogram (17), tractography (18), resting state functional imaging (19, 20), and anatomical morphometry (21, 22). Though results vary across modalities, one consistent result is a reduction in small world index (SWI) (19, 21). Increased clustering, a measure of segregation, has been shown with cortical thickness (21), gyrification (22), and resting state functional connectomes (20), while decreased global efficiency, a measure of integration, has been seen with resting state functional connectivity (20) and tractography connectomes (18). In addition, a modulatory effect of treatment with antipsychotic drug (APD) has been observed in resting state functional connectome (20).

The effects of APD on gyral patterning are relevant to investigation because there is evidence that APDs modulate gray matter tissue. Prolonged use and greater dosage of ADPs are linked to reduced gray matter volume (23, 24), although increased striatal volume has been reported and found to correlate with better treatment response (25). There is evidence that 2nd generation APDs are associated with less cortical thinning than first generation APDs (26–29), suggesting they could mitigate the progressive loss of gray matter tissue. A recent longitudinal study in medication naïve patients reported a significant increase in gray matter volume after 8 weeks of APD treatment (30). Known associations between greater duration of untreated psychosis and worse outcome (31) appear to also suggest that APD treatment early in the illness could mitigate a possible neurobiological process underlying clinical deterioration (32). Meanwhile, gyrification based morphological covariance is of interest because it has been shown to be both a better indicator of neurodevelopmental progression and a more effective measure when controlling for brain size differences than cortical thickness (12, 33). This arguably makes the use of local gyrification index (34) a good method for investigating subtle neurodevelopmental alterations.

The goal of the present study was to evaluate morphological covariance using a measure of cortical gyrification as well as the effect of a short trial of APD on this measure in unmedicated patients with schizophrenia (SZ). In this study we used a prospective design where unmedicated patients with schizophrenia were scanned prior to treatment and again after 6 weeks of treatment with risperidone, a frequently used 2nd generation APD. To control for the effect of time, we scanned a matched group of healthy controls (HC) 6 weeks apart. The aims of this study were two-fold: (1) Evaluate, in unmedicated patients, the structural covariance of local gyrification index using graph theory measures of integration and segregation, (2) investigate the effect of a short antipsychotic treatment on graph theory measures of integration and segregation. On the basis of the existing literature, we hypothesized that, in unmedicated patients, we would observe greater segregation and reduced integration measures of local gyrification index when compared with matched HC, and that a short course of APD treatment would modify these measures toward more normal patterns.

## Methods

### Subjects

Thirty-seven unmedicated patients with schizophrenia or schizoaffective disorder (*n* = 37, 22 medication naïve and 12 patients with prior APD exposure), were recruited from the emergency room, inpatient units, and outpatient clinics at the University of Alabama at Birmingham (UAB). Average illness onset was 22.08 years and average illness duration was 15 years with a median length of 18.5 years (Table 1). Twenty three HC (*n* = 23) matched on age, gender, smoking, and parental socioeconomic status (SES) were recruited by advertisements. Exclusion criteria were major neurological or medical conditions, a history of head trauma with loss of consciousness, substance use disorders (excluding nicotine) within 6 months of imaging, pregnancy or breastfeeding, and MRI contraindications. In addition, HC with a lifetime history of a psychiatric disorder or a family history of a psychiatric illness in a first-degree relative were excluded. Approval for this study was given by the UAB Institutional Review Board and written informed consent was obtained prior to enrolment and after subjects were deemed to have capacity to provide consent (35).

**Table 1:** Demographics, clinical measures, and covariates^a^.

	**SZ (*n* = 34)**	**HC (*n* = 23)**	***t*/χ^2^**	***p-*value**
Gender (%male)	73.5	82.6	0.642	0.423
Age	28.32 (9.42)	27.48 (9.63)	−0.330	0.743
Socioeconomic status^b^	5.88 (5.06)^c^	4.65 (3.97)	12.849	0.303
Smoking (packs per day)	0.37 (0.49)	0.18 (0.41)	−1.564	0.124
Illness duration (years)^d^	15.00 (8.45) (*n* = 12)			
Illness onset (years)^d^	22.08 (3.12)			
APD naïve (yes/ no)	22/12			
eTIV^e^	1593.70 (182.36)	1658.79 (230.71)	1.187	0.240
SNR	19.93 (1.98)	20.50 (2.07)	1.069	0.290
BPRS^f^	Baseline	Week 6		
Total	50.91 (9.65)	32.50 (10.45)	7.545	<0.001
Positive	10.44 (3.47)	5.18 (2.41)	7.277	<0.001
Negative	7.44 (3.16)	5.85 (2.74)	2.215	0.030

*SZ, schizophrenia; HC, healthy controls; APD, antipsychotic drug; eTIV, Estimated total intracranial volume; SNR, signal to noise ratio; BPRS, Brief Psychiatric Rating Scale*.

a *Mean (SD) unless indicated otherwise*.

b *Parental socioeconomic ranks determined from Diagnostic Interview for Genetic Studies (1–18 scale), higher rank (lower numerical value) corresponds to higher socioeconomic status*.

c *Data not available for 2 SZ subjects, n = 32*.

d *Includes only patients who are not antipsychotic naïve (n = 12), illness duration since first diagnosis*.

e *Estimated total intracranial volume (eTIV) in cm^2^*.

f *BPRS (1–7 scale); positive (conceptual disorganization, hallucinatory behavior, and unusual thought content); negative (emotional withdrawal, motor retardation, and blunted affect)*.

### Study Design and Clinical Assessments

Unmedicated SZ (either antipsychotic medication-naïve, or off antipsychotic medications for at least 2 weeks), were enrolled in a 6-weeks trial of risperidone using a flexible dosing regimen. Risperidone was started at 1–3 mg and titrated in 1–2 mg increments, average Risperidone dosage was 4 mg (standard deviation 1.52); pill counts were done to monitor compliance. Use of concomitant psychotropic medications was permitted as clinically indicated. Concomitant medications and number of patients taking them included benztropine (19), fluoxetine (2), trazodone (2), amitriptyline (1), valproic acid (1), clonazepam (1), mirtazapine (1), desvenlafaxine (1), sertraline (1). One patient did not have medication information.

Diagnoses were established by review of medical records, the Diagnostic Interview for Genetic Studies (DIGS) (36), and consensus of two board certified psychiatrists (ACL and NVK). At each visit, symptom severity was assessed using the Brief Psychiatric Rating Scale (BPRS) (37), and its positive and negative symptom subscales.

### MRI Acquisition

Structural scans were obtained prior to treatment (off medication), and after 6 weeks of treatment. HC were also scanned twice, 6 weeks apart. Imaging data was collected on a head-only 3T MRI equipped with a circularly polarized transmit/receive head coil that was used for all imaging (Magnetom Allegra, Siemens Medical Solutions). The three-dimensional T1-weighted magnetization prepared rapid acquisition gradient echo sequence (MPRAGE) was used for structural acquisition (TR/TE/TI = 2,300/3.93/1,100 ms, flip angle = 12°, 256 × 256 matrix, 1 mm isotropic voxels).

### Data Pre-processing and Quality Control

The cortical surface of each structural image was reconstructed and automatically parcellated into atlas based regions (38) using FreeSurfer 5.3 (39). FreeSurfer's QA Tools (https://surfer.nmr.mgh.harvard.edu/fswiki/QATools) was then used to assess quality of each reconstructed data set by (1) verifying the presence of all appropriate output files and the correct order of processing steps, (2) measuring signal to noise ratio and white matter intensity, and (3) manual inspection of detailed snapshots of each subject's preprocessed structural image. Scans with poor initial data quality were manually corrected or, if correction was unsuccessful, excluded from final analysis. Three SZ were removed from analysis due to irreparable imaging artifacts resulting in 34 SZ and 23 HC retained for final analysis. After quality assurance, the remaining images were reprocessed using the FreeSurfer longitudinal stream (40). This adjusted the original pipeline outputs to unbiased within-subject template spaces, in order to increase power and reliability of subsequent analysis.

### Local Gyrification Index

Local gyrification index values were computed across the cortical surface by generating an outer surface mesh enclosing and overlapping the reconstructed cortical surface, producing a 25 mm spherical region of interest (ROI) at each vertex of the outer surface mesh and calculating a ratio of cortical surface area to outer surface area for each ROI. The values were then propagated to corresponding cortical surface vertices resulting in a global heat map of local gyrification index values across the reconstructed cortical surface for each participant at each time point (34).

### Demographic and Regression Analysis

Group differences for age, gender, smoking status, parental socioeconomic status (SES), signal to noise ratio (SNR), and estimated total intracranial volume (eTIV) (41) were assessed in SPSS via independent samples *t*-tests or chi-square tests where appropriate. Because of functional, structural, and connectivity lateralization asymmetries in SZ (42–44), we calculated local gyrification index for each hemisphere separately. These values were used as dependent variables in 2 separate analyses for each hemisphere using a 2 (baseline vs. week 6) × 2 (HC vs. SZ) mixed analysis of covariance (ANCOVA) model. Age, gender, and eTIV were included as covariates of no interest.

### Graph Analyses

Local gyrification index connectomes were computed in Matlab using the Graph Analysis Toolbox (GAT) (45). To define ROI's, we employed the Desikan-Killiany atlas, a gyral based atlas (38) that has been widely used to investigate cortical morphometry in SZ (11, 14, 22, 46–48). Average local gyrification index was calculated for 68 (34 per hemisphere) ROIs and were entered into a 68 × 68 Pearson's correlation matrix that was adjusted for age, gender, and eTIV. This was done for each group at baseline and at 6 weeks. The longitudinal GAT pipeline does not use a single thresholding coefficient or a range of coefficients and removes only negative connections when constructing each network. Longitudinal comparisons were standardized using weighted networks for analyses.

We calculated several graph metrics. First, we calculated the SWI, a general representation of network connectivity indicating the relationship between segregative and integrative network properties. Biological networks are expected to have a SWI > 1 implying that connectivity within the network is not random and retains a high level of specialization. Measures of network integration included shortest path length (the smallest number of edges between any two nodes) and global efficiency (inverse of the average path length). Measures of network segregation included clustering coefficient (ratio of the number of actual connections between neighboring nodes over the number of possible connections between neighboring nodes) and local efficiency (the inverse of the shortest path connecting all neighboring nodes of a given node). Other measures included were centrality termed betweenness (the number of shortest path lengths that traverse through a given node) and modularity, which indicates how many highly clustered subnetworks exist within the larger network. Modularity was calculated using a Newman's optimization algorithm (49).

To test for significant time by group interaction effects of each topological measure, 20 Null-hypothesis networks were generated for each matrix. These null graphs were pseudo-randomly generated by algorithmically accounting for the distributional properties of each originating matrix (50). Following this, 95% null confidence intervals were computed using a 1,000 repetition non-parametric permutation test on each of the 20 generated null graph sets. The permutation tests would retain the local gyrification index values for each ROI but randomize those values across individuals at each repetition. The time by group difference *p*-values of each topological measure from the sample data were then compared to the corresponding 95% null distribution to determine significance. *Post-hoc* comparisons (examining effect of group and examining effect of time) were conducted for any topological measures that showed a significant group by time interaction. This analysis was similar to the longitudinal graph analysis described above, but because the longitudinal comparison was analyzed using a weighted approach, using the same approach for each *post-hoc* test could have potentially confounded results by differences in minimum graph density (and thus changes in connectome properties) in each comparison. To correct for this, we specified a standard range of density coefficient thresholds for all *post-hoc* comparisons (0.12, the maximum minimum density produced across all four comparisons, to 0.5 in increments of 0.02). To further standardize results, significance of *post-hoc* differences were calculated based on area under the curve (AUC) comparisons limiting sensitivity to the thresholding process.

## Results

No significant group differences were observed for sex, age, parental SES, packs per day, eTIV, or SNR (all *p* > 0.05). BPRS scores significantly decreased after 6 weeks of treatment with risperidone (Table 1).

For measures of local gyrification index, the main effect of group was significant for each hemisphere [left: *F*_1, 51_ = 15.63 *p* < 0.001; right: *F*_1, 51_ = 15.58 *p* < 0.001; Table 2]. Overall local gyrification index was greater for HC than SZ in both hemispheres.

**Table 2:** Mixed MANCOVA results for LGI per hemisphere.

	**SZ (*n* = 34)**		**HC (*n* = 23)**		**Left**		**Right**	
	**Baseline**	**Week 6**	**Baseline**	**Week 6**	***F***	***P***	***F***	***p***
	**M (SD)**	**M (SD)**	**M (SD)**	**M (SD)**				
**LGI**								
Left Hemisphere	2.946 (0.105)	2.940 (0.101)	3.072 (0.156)	3.065 (0.148)	–	–	–	–
Right Hemisphere	2.958 (0.111)	2.956 (0.112)	3.077 (0.146)	3.073 (0.148)	–	–	–	–
Group	–	–	–	–	15.636	<0.001	15.584	<0.001
Time	–	–	–	–	0.907	0.345	0.106	0.746
Time × Group	–	–	–	–	0.074	0.786	0.084	0.773

*SZ, schizophrenia; HC, healthy controls; M, mean; SD, standard deviation; LGI local gyrification index. Covariates accounted for include: age, gender, and estimated total intracranial volume. Repeated measures interactions and between subjects effects not included*.

### Graph Analysis

**Figure 1:**
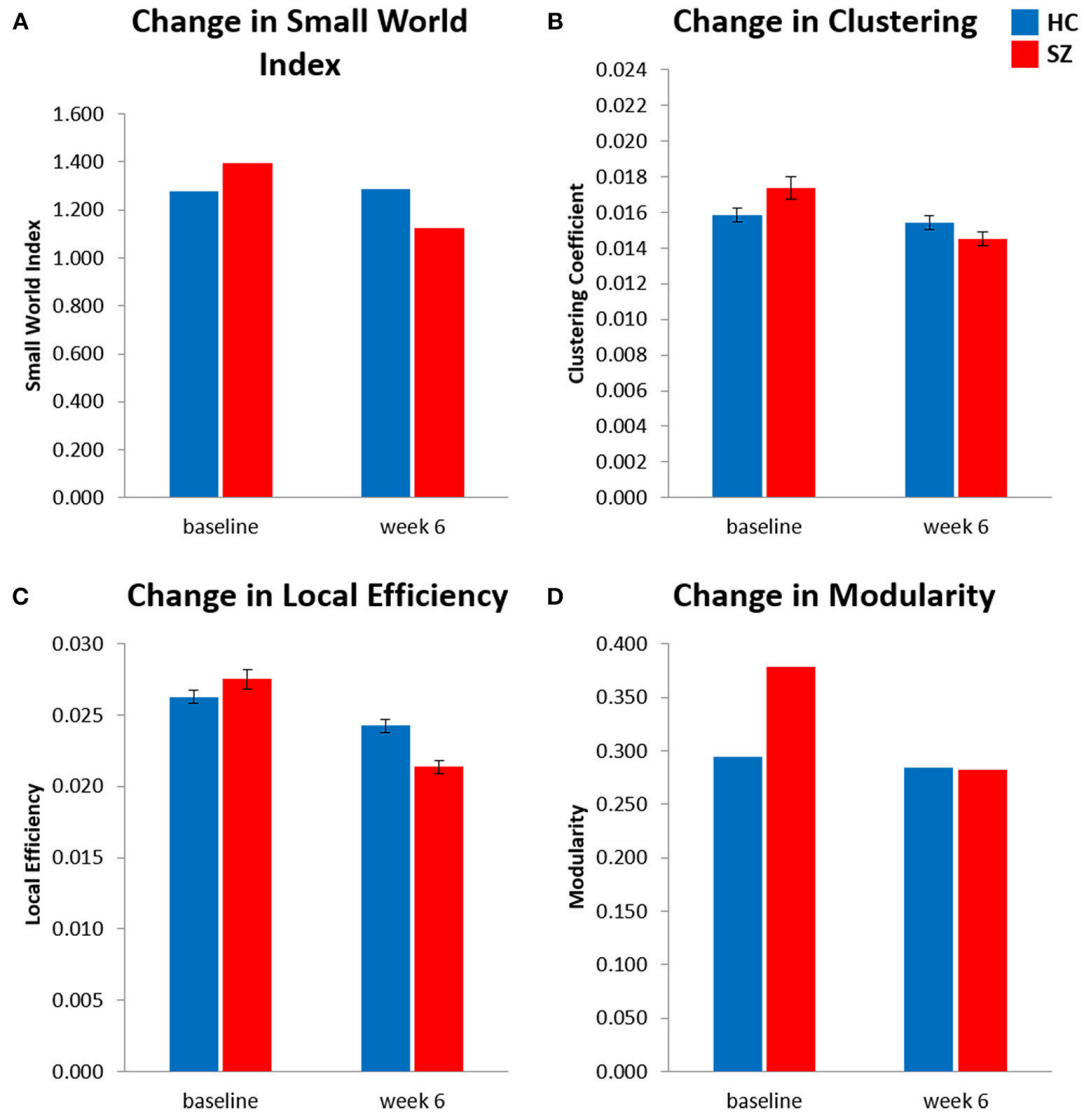
Longitudinal local gyrification index based connectome results showed significant time (baseline vs. week 6) × group [healthy control (HC) vs. patients with schizophrenia (SZ)] interactions for **(A)** small world index, **(B)** global clustering coefficient, **(C)** global local efficiency, and **(D)** modularity. Analysis controlled for age, sex, and estimated total intracranial volume. Significance is based on 95% confidence interval (*p* < 0.05). Error bars show standard error for clustering coefficient and local efficiency only, small world index and modularity were calculated as singular values for each connectome. SZ, schizophrenia; HC, healthy controls.

The results of the longitudinal local gyrification index based connectome analysis showed that all graphs (HC and SZ at baseline and week 6) had SWI > 1, indicating that all graphs presented with small-world organization (Table 3). Furthermore, longitudinal SWI comparisons showed a significant group by time interaction (*p* = −0.035; Figure 1A). *Post-hoc* tests for SWI were not significant (Table 4). Measures of integration (path length and global efficiency) did not show significant group by time interactions, nor did betweenness, the measure indicating network centrality (Table 3). However, group by time interactions of segregation measures were significant (clustering coefficient: *p* = 0.002; local efficiency: *p* = 0.016; Figures 1B,C). *Post-hoc* analyses show that clustering and local efficiency differed between groups at baseline (clustering coefficient: *p* = 0.029; local efficiency: *p* = 0.018), but not at week 6 (Table 4). Modularity also had a significant group by time interaction (*p* = 0.027; Figure 1D). Similar to clustering and local efficiency, *post-hoc* modularity results show only group differences at baseline (*p* = 0.002) (Table 4). Coefficient values for the topological measures of each group at both time points and corresponding *p*-values for each interaction test can be found in Table 3.

**Table 3:** Longitudinal topological results.

	**SZ (*n* = 34)**		**HC (*n* = 23)**		***p*-value**
	**Baseline**	**Week 6**	**Baseline**	**Week 6**	
Small-world index	1.394	1.124	1.278	1.285	−0.035^*^
Path length	0.008	0.004	0.007	0.005	−0.106
Global efficiency	0.030	0.026	0.030	0.028	0.137
Clustering coefficient	0.017	0.015	0.016	0.015	−0.002^*^
Local efficiency	0.028	0.021	0.026	0.024	−0.016^*^
Betweeness	175.3	218.0	172.4	201.3	0.133
Modularity	0.379	0.282	0.294	0.284	−0.027^*^

*SZ, schizophrenia; HC, healthy controls. P-values based on 95% confidence interval. Results corrected for age, gender, and estimated total intracranial volume*.

* *Indicates significance p < 0.05*.

**Table 4:** Post hoc area under the curve comparisons.

	**Within group;**		**Between group;**	
	**Week 6 > Baseline**		**SZ > HC**	
	**HC**	**SZ**	**Baseline**	**Week 6**
Small world index	0.319	−0.073	0.064	−0.151
Path length	0.332	0.487	0.098	0.210
Global efficiency	−0.323	−0.495	−0.074	−0.222
Clustering coefficient	0.247	0.454	0.029^*^	0.282
Local efficiency	0.293	−0.477	0.018^*^	0.371
Betweeness	0.335	0.485	0.100	0.209
Modularity	0.172	−0.194	0.002^*^	0.296

*SZ, schizophrenia; HC, healthy controls. Numbers are comparison p-values based on 95% confidence interval. Results corrected for age, gender, and estimated total intracranial volume*.

* *Indicates significance p < 0.05*.

## Discussion

The purpose of this longitudinal, prospective study was to examine cortical gyrification covariance using graph theory measures in unmedicated patients with schizophrenia, and to evaluate the effect of a short trial of APD on these measures. At baseline, measures of segregation were significantly greater in SZ compared to HC. After 6 weeks of treatment, there were no significant differences in segregation measures between SZ and HC. These results indicate that abnormal cortical topography in SZ is present at baseline likely indexing neurodevelopmental trajectory deviations and is modified by short term APD treatment reflective of decreases in hyper-specialization in network connectivity.

Results of the LGI time by group interaction indicated that, overall, HC showed greater LGI than SZ in both hemispheres. These findings are in contrast to previous reports in the high-risk for psychosis (9, 51) and first-episode psychosis populations (52–55). This discrepancy could result from several factors. With the exception of Sasabayashi (51, 54) these studies did not use local gyrification index as a measure to assess cortical folding. Harris et al. (52) used a hand traced method, Zuliani et al. (55) and Harris et al. (9) used Automated GI (56), while Schultz et al. (53) measured mean curvature. In addition, our analysis used average local gyrification index values per hemisphere per participant. In contrast to this approach, Zuliani et al. (55) and Harris et al. (9) measured prefrontal gyrification, Harris et al. (52) analyzed cortical folding per lobe, and Sasabayashi (51, 54) and Schultz et al. (53) compared gyrification at a voxel-wise level.

In unmedicated patients with schizophrenia, we found increased network segregation (high clustering and local efficiency). Increases in clustering coefficient have also been seen in cortical thickness (21) and functional resting state (20) connectomes, indicating that abnormally high levels of local specialization are seen across modalities in SZ. Cross-modal comparison of connectivity supports concordance between functional and structural connectomes (57). In a local gyrification index connectome study where patients were evaluated prior to treatment and treatment response was determined subsequently, Palaniyappan et al. (22) demonstrated that those who subsequently would show a poor response to treatment had, before treatment, higher level of segregation and lower level of integration compared to good responders and controls (22). These data suggest that those with subsequent poor response to treatment had a higher burden of developmental insult. Our small sample did not allow us to dichotomize the patients into good and poor responders; however, the prospective design of our study allowed us to focus on the effect of medication. In our patients, segregation measures normalized to HC levels after treatment. Post medication reduction in clustering coefficient was also seen in the resting state connectome comparisons of Hadley et al. (20). On the other hand, we did not detect significant alterations in integration measures at baseline or after treatment. We also observed increased baseline modularity, an indicator of the magnitude of network division into specialized groups, in SZ compared to HC. Networks with high modularity are heavily connected within modules, but sparsely connected between modules. In addition to decreased integration Palaniyappan et al. (22) showed decreased modularity in the poor treatment responders compared to the good responders and controls.

In medication naïve patients with schizophrenia, abnormal gyrification covariance is likely indexing deviations from normal neurodevelopment trajectory. Much is now known about pre and perinatal risk factors in schizophrenia, such as prenatal exposure to viral infection and nutritional deficiencies, sequelae of hypoxia, and other obstetric hazards (58), these risk factors could derail the trajectory of brain development at various stages of prenatal ontogenesis, such as neuronal birth, proliferation, migration, and differentiation (59). Genes have also been shown to influence the coordinated growth of spatially separated regions (60), and some of the genes and genetic variants associated with schizophrenia are known to impact brain development (61). Speculatively, aberrant synaptic pruning during adolescence as proposed by Feinberg (62), as well as exposure to postnatal risk factors, such as childhood adversities or cannabis use (58) could affect levels of gyrification covariance later in life. In addition, in those patients who had been previously exposed to APDs, additional factors such as illness chronicity and cumulative exposure to APDs could have affected gyrification covariance as well.

In schizophrenia, gray matter reductions are already observed at illness onset (63), in those at high risk for psychosis (64), as well as in the relatives of subjects with schizophrenia (65). It is now well-established that these alterations become more pronounced and extensive over time (23, 66, 67). Contributors to these gray matter reductions include poor clinical outcome (68, 69), duration of clinical relapses (70), cannabis use (71) and genetic liability (72). The extent to which antipsychotic medications contribute to those deficits is still debated; this is in part because of the difficulty distinguishing between illness progression and the effect of medication (73). Prolonged use and greater dosage of APDs are linked to reduced gray matter volume (23, 24), although increased striatal gray volume has been reported and found to correlate with better treatment response (25). There is evidence that 2nd generation APDs are associated with less cortical thinning than first generation APDs (26–29), suggesting they could mitigate the progressive loss of gray matter tissue. A recent longitudinal study in medication naïve patients reported a significant increase in gray matter volume after 8 weeks of treatment (30). Known associations between greater duration of untreated psychosis and worse outcome (31) appear also to suggest that APD treatment early in the illness could mitigate a possible neurobiological process underlying clinical deterioration (32).

APDs have been shown to have trophic effect on brain tissue, such as an increase in the level of synaptic proteins and the promotion of dendritic growth (74). One of these proteins, brain derived neurotrophic factor (BDNF) which is stored and released by glutamatergic neurons, is an important regulator of synaptic transmission. BDNF is also essential to synaptic plasticity and helps protect against apoptosis (75, 76). In addition, BDNF is associated with increases in spine density levels (77). A large meta-analysis that included over 7,000 subjects by Fernandes et al. (78) shows that SZ is associated with lower levels of BDNF and that these levels increased with APD treatment. There is precedence that 2nd generation antipsychotics in particular help reverse, or at least alleviate, dendritic atrophy of the outer layers of the cortex (79). Given that volume change in SZ appears to be the result of a decrease in neuropil rather than cell death, it could be argued that treatment with risperidone, a prototypical 2nd generation APD, could modulate neuronal growth, and while it is doubtful that a short term trial with APD would modify gyrification patterns, this growth could rewire neuronal signaling in a way that can influence structural network organization. We previously reported changes in resting state functional connectivity (2, 80) and resting state functional covariance reflective of decrease segregation and increase integration (20) following APD treatment. We speculate that the trophic effects of APDs on brain tissue support changes in structural and functional network organization, promoting a better connected brain and, possibly, providing a therapeutic effect.

### Strength and Limitations

To avoid confounding medication effects and minimize data variance, we only enrolled unmedicated SZ, matched groups on several key factors, and used a rigorous longitudinal design with a single antipsychotic medication. Also, we attempted to control for the effect of time by scanning a HC group 6 weeks apart. On the other hand, our small sample likely limited our ability to replicate or detect other important findings.

## Conclusions

Abnormal cortical topography is detected in schizophrenia, likely indexing neurodevelopmental trajectory deviations. In addition, cortical topography is modified by short term APD treatment reflective of decreases in hyper-specialization in network connectivity. We speculate that changes in the structural organization of the brain is achieved through the trophic effects that APDs have on brain tissue, thus promoting better brain connections and, possibly, a therapeutic effect.

## Author Contributions

AL was responsible for the study concept and design. AL and DW supervised the study. EN and NK conducted the statistical analyses and drafted the manuscript. All authors contributed to acquisition, analysis and interpretation of the data, and critically reviewed the content of the manuscript for intellectual content. AL is the guarantor.

### Conflict of Interest Statement

Medication for this study was donated to AL by Janssen Pharmaceuticals, Inc. AL received an investigator initiated grant from Janssen Pharmaceuticals, Inc. The remaining authors declare that the research was conducted in the absence of any commercial or financial relationships that could be construed as a potential conflict of interest.
